# Neuroprotective Effects of Citicoline in *in Vitro* Models of Retinal Neurodegeneration

**DOI:** 10.3390/ijms15046286

**Published:** 2014-04-14

**Authors:** Andrea Matteucci, Monica Varano, Lucia Gaddini, Cinzia Mallozzi, Marika Villa, Flavia Pricci, Fiorella Malchiodi-Albedi

**Affiliations:** 1 Department of Cell Biology and Neuroscience, Istituto Superiore di Sanità, Viale Regina Elena, 299, Rome 00161, Italy; E-Mails: andrea.matteucci@iss.it (A.M.); lucia.gaddini@iss.it (L.G.); cinzia.mallozzi@iss.it (C.M.); flavia.pricci@iss.it (F.P.); 2 GB Bietti Eye Foundation IRCCS, Via Livenza, 3, Rome 00198, Italy; E-Mails: m.varano@mclink.it (M.Va.); marean@inwind.it (M.Vi.)

**Keywords:** citicoline, glaucoma, excitotoxicity, apoptosis, diabetic retinopathy, primary retinal cultures, neuroprotection

## Abstract

In recent years, citicoline has been the object of remarkable interest as a possible neuroprotectant. The aim of this study was to investigate if citicoline affected cell survival in primary retinal cultures and if it exerted neuroprotective activity in conditions modeling retinal neurodegeneration. Primary retinal cultures, obtained from rat embryos, were first treated with increasing concentrations of citicoline (up to 1000 μM) and analyzed in terms of apoptosis and caspase activation and characterized by immunocytochemistry to identify neuronal and glial cells. Subsequently, excitotoxic concentration of glutamate or High Glucose-containing cell culture medium (HG) was administered as well-known conditions modeling neurodegeneration. Glutamate or HG treatments were performed in the presence or not of citicoline. Neuronal degeneration was evaluated in terms of apoptosis and loss of synapses. The results showed that citicoline did not cause any damage to the retinal neuroglial population up to 1000 μM. At the concentration of 100 μM, it was able to counteract neuronal cell damage both in glutamate- and HG-treated retinal cultures by decreasing proapoptotic effects and contrasting synapse loss. These data confirm that citicoline can efficiently exert a neuroprotective activity. In addition, the results suggest that primary retinal cultures, under conditions inducing neurodegeneration, may represent a useful system to investigate citicoline neuroprotective mechanisms.

## 1. Introduction

Citicoline (cytidine-5′-diphosphocholine) is an essential precursor in the synthesis of phosphatidylcholine, a component of cell membranes. Several experimental studies *in vitro* and *in vivo* have suggested that citicoline is neuroprotective. In particular, citicoline has been found to efficiently counteract neuronal cell damage in animal models of cerebral ischemia [1]. In human studies, recent data suggest that administration of citicoline may slow down certain neurodegenerative diseases. In mild vascular cognitive impairment, oral citicoline significantly improved the Mini-Mental State Examination score and favorably influenced patients’ mood [2]. In addition, in sub-acute ischaemic cerebrovascular disease, administration of citicoline has been found to improve functional rehabilitation and reduce the burden of care [3].

Fewer studies have investigated on the possible use of citicoline in the treatment of neurodegenerative diseases of the retina. Citicoline administration may efficiently reduce signs of the disease in glaucoma, where the retina undergoes neurodegenerative changes. In glaucoma patients, oral or intramuscular administration of citicoline was associated with an improved retinal and visual pathway functions [4–6]. Moreover, in patients with progressive glaucoma, oral citicoline was recently found to slow down glaucomatous rates of progression [7].

Here we have tested citicoline neuroprotection *in vitro* in conditions relevant to neuroretinal degeneration: glutamate-induced excitotoxicity, which is considered of pathophysiological relevance in glaucoma, and High Glucose (HG)-induced neurotoxicity, characteristic of diabetic retinopathy. *In vitro*, citicoline has already been shown to protect against HG neurotoxicity in retinal explants [8]. In addition, it has proven efficient in preventing excitotoxic cell damage in cerebellar granule cells [9] and motor neurons [10]. The purpose of this study was to assess citicoline neuroprotective ability against both neurotoxic conditions *in vitro* using primary retinal cultures, which can differentiate *in vitro* to produce mature neurons and Müller glia. Our results support the use of this system as a valuable tool to investigate the neuroprotective mechanisms of probes, such as citicoline, relevant to retinal neurodegeneration.

## 2. Results and Discussion

### 2.1. Citicoline Is Well Tolerated in Primary Retinal Cultures

**Figure 1.**
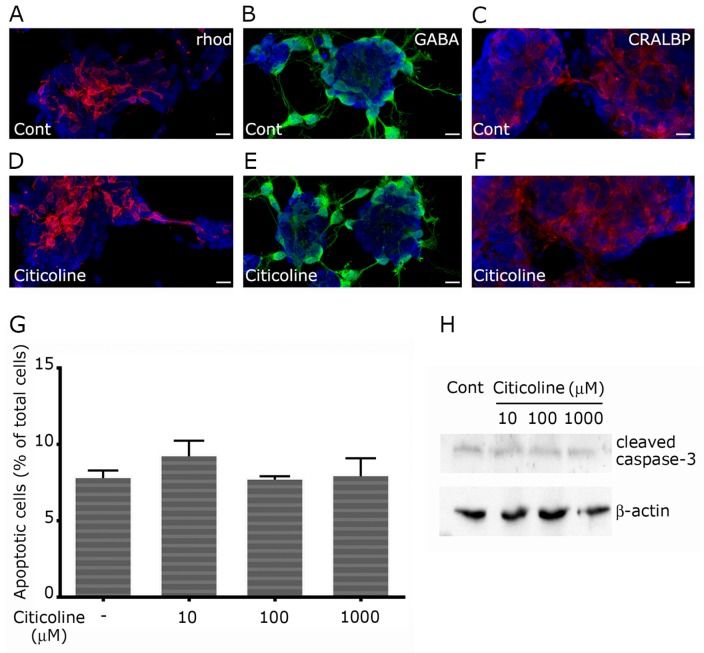
Citicoline treatment does not induce cytotoxic effect in primary retinal cultures. Primary retinal cultures, treated or not with 100 μM citicoline for 96 h, were immunolabeled with antibodies against rhodopsin (**A**,**D** rhod), GABA (**B**,**E**) and CRALBP (**C**,**F**), in order to evidence photoreceptors, GABAergic neurons and Müller glia, respectively. Cell nuclei were counterstained with Hoechst 33258. Antibody immunoreactivity was unaffected in citicoline-treated samples, showing that the agent does not influence cell composition and differentiation in primary retinal cultures (**D**–**F**); Apoptosis was evaluated by TUNEL assay. Apoptotic nuclei were quantified as percentage of TUNEL-positive nuclei over total nuclei. Bars represent the mean ± SEM of at least five independent experiments (**G**); Absence of proapoptotic effects after citicoline treatment is also shown by unchanged levels of activated cleaved caspase 3 (**H**). Whole cell lysates were prepared from primary retinal cultures. Equal amounts of total protein from each lysate were resolved on 15% SDS-PAGE and transferred to nitrocellulose membranes. Membranes were probed with anti-cleaved caspase 3. β-actin levels were used as control of protein loading. Blots are representative of three independent experiments. Bars = 20 μm.

**Figure 2.**
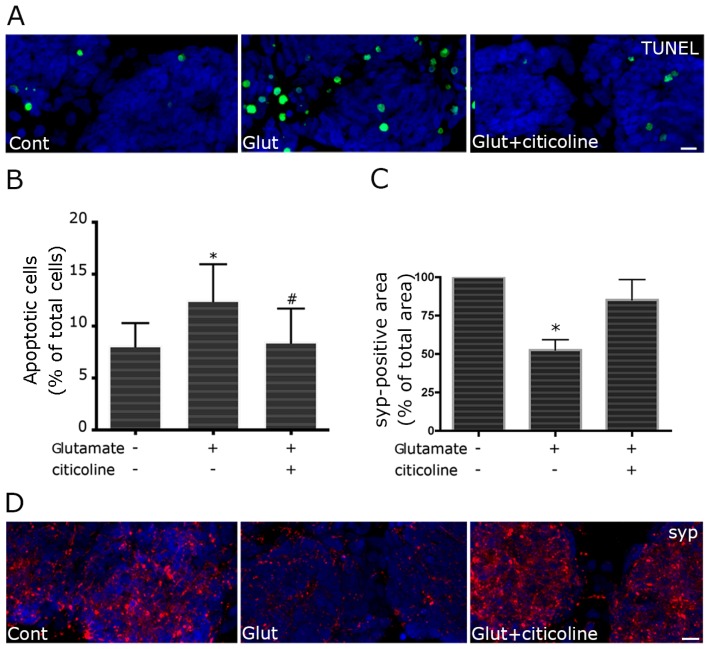
Citicoline treatment protects retinal cells against glutamate-induced apoptosis and synaptotoxicity. To evaluate protective ability of citicoline against excitotoxicity, primary retinal cultures were treated with 100 μM citicoline 24 h before excitotoxic insult, induced with exposition to 100 μM glutamate for 25 min. The medium was then replaced with fresh Minimum Essential Medium/10% Fetal Calf Serum (MEM/FCS) and the cells were fixed after 24 h. Apoptosis was evaluated by TUNEL assay (**A**,**B**); Synapses were evidenced by immunolabeling of synaptophysin (**C**,**D**). In glutamate-treated retinal cultures, the increase in apoptotic nuclei (green) is counteracted by citicoline treatment (**A**); TUNEL-positive nuclei were counted and apoptosis was expressed as percentage of apoptotic cells over total cells. Bars represent the mean ± SEM of at least five independent experiments (**B**); Citicoline treatment significantly reduces the apoptotic rate in glutamate-treated cells. * *p <* 0.05 *vs.* control; # *p <* 0.05 *vs.* glutamate-treated cells, Wilcoxon matched pairs test. For morphometric analysis of synaptophysin immunostaining, at least eight fields were captured for each culture. The density of synaptic puncta was measured as synaptophysin-positive area in each selected field, applying the same threshold parameters to all images. Values were pooled to obtain a single mean value for each condition. Significantly lower positivity for synaptophysin is observed after glutamate treatment. The decrease is reversed in the presence of citicoline (**C**). * *p <* 0.05, Mann-Whitney *U* test. In (**D**), representative images are shown (syp, red). Bars = 20 μm.

**Figure 3.**
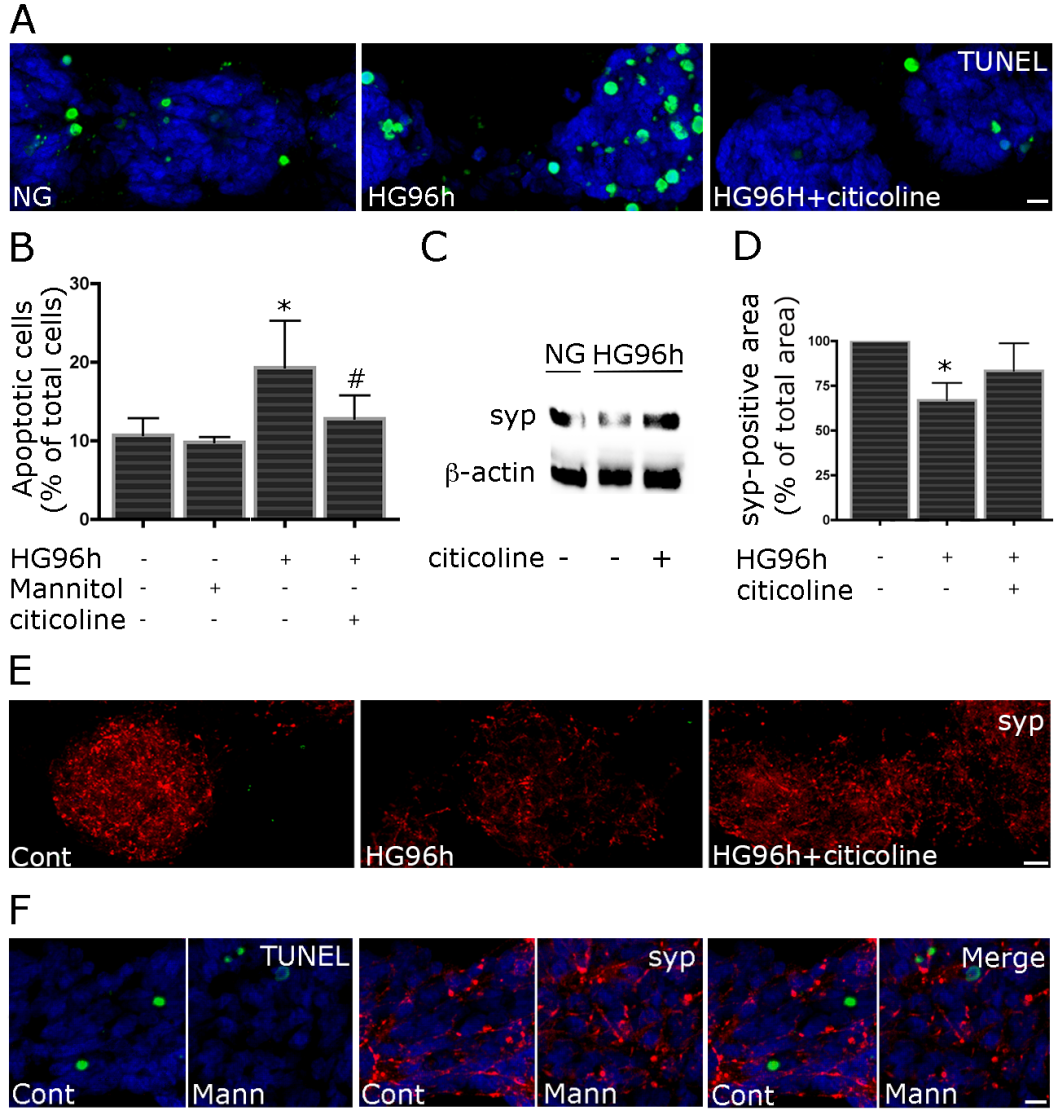
Citicoline treatment protects retinal cells against HG-induced apoptosis and synaptotoxicity. To evaluate protective ability of citicoline against the diabetic milieu, primary retinal cell cultures were treated with daily changes of HG for 96 h, in the presence or not of 100 μM citicoline. Controls included treatment with iso-osmolar mannitol. Apoptosis was evaluated by TUNEL assay. In HG-treated retinal cultures, the increase in apoptotic nuclei (green) is counteracted by citicoline treatment (**A**); TUNEL-positive nuclei were counted and apoptosis was expressed as percentage of apoptotic cells over total cells. Bars represent the mean ± SEM of at least five independent experiments (**B**); Citicoline treatment significantly reduces the apoptotic rate in HG-treated cells. *p < 0.05 *vs.* control; # *p <* 0.05 *vs.* HG-treated cells, Wilcoxon matched pairs test. Primary retinal cultures were analyzed for Western Blotting (WB) Equal amounts of total protein from each lysate were resolved on 10% SDS-PAGE and transferred to nitrocellulose membranes. Membranes were probed with anti-synaptophysin and β-actin as a control of protein loading. Blot is representative of three independent experiments (**C**); HG decreased synaptophysin levels, while in the presence of citicoline the levels were similar to control cultures. For morphometric analysis of synaptophysin immunostaining, at least eight fields were captured from each culture. The density of synaptic puncta was measured as a synaptophysin-positive area in each selected field, applying the same threshold parameters to all images. Values were pooled to obtain a single mean value for each condition. Significantly lower positivity for synaptophysin was observed after HG treatment. The decrease is reversed in the presence of citicoline (**D**), * *p <* 0.05, Mann-Whitney *U* test; In (**E**), representative images are shown (syp, red). Treatment with iso-osmolar mannitol did not induce increase in TUNEL positive nuclei or reduce synaptophysin immunostaining. In (**F**), representative images are shown. Bars = 20 μm.

Dissociated cultures from embryonic rat retina are composed of mixed neuronal and glial cell population. Neuronal cells, composed mostly of rhod-positive photoreceptors (Figure 1A), forming rosette-like structures, and GABAergic neurons (Figure 1B), were interspersed among Cellular Retinaldehyde-Binding Protein (CRALBP)-positive Müller glia (Figure 1C). At 9–10 days *in vitro* (DIV), neuronal cells were well differentiated, as shown by the formation of synaptophysin-positive synapses (Figures 2D and 3E).

First of all, we investigated if 100 μM citicoline could induce cell damage in a specific cell type. As shown in Figure 1D–F, both glial or neuronal cell components were well preserved in citicoline-treated cultures, with no evidence of selective toxicity after treatment. In addition, the neuronal cell types that predominate in the cell cultures, *i.e*., photoreceptors and GABAergic neurons, were similarly represented in both control and citicoline-treated cell cultures. We then verified if treatment with citicoline induced cell damage in our *in vitro* system in terms of increased apoptotic rate. In primary retinal cultures at DIV 6, treated with increasing concentrations of citicoline (10, 100, 1000 μM) for 96 h, apoptosis was analyzed evaluating TUNEL-positive nuclei (Figure 1G) and caspase 3 activation (Figure 1H). Up to 1000 μM citicoline, no differences were observed for both the examined parameters in treated cultures, with respect to control cultures. Our results confirmed that citicoline is well-tolerated when administered to retinal cultures, since it does not modify the apoptotic trend, evaluated in terms of TUNEL-positive nuclei and caspase activation, and does not affect cell culture composition, as both neuronal and glial components were analogously represented in control and treated cultures. These results confirm that citicoline is not harmful for retinal neuroglial cells *in vitro*, in line with the good tolerability profile exhibited by citicoline in clinical studies.

### 2.2. Citicoline Protects against Excitotoxic Cell Damage

To determine if citicoline showed neuroprotective ability against retinal neurodegeneration, we set up models of both glutamate-induced excitotoxicity and HG-promoted neuronal cell damage. Excitotoxic neuronal damage was examined in a “delayed” excitotoxic protocol, which characteristically triggers an apoptotic pathway of cell death [11]. In primary retinal cell cultures, this treatment induced a mild, though statistically significant, increase in TUNEL-positive nuclei (Figure 2A,B). Such a moderate neurotoxic effect is probably more appropriate to mimic a slowly progressing, neurodegenerative disease such as glaucoma [12]. In the presence of 100 μM citicoline, a significant reduction of apoptotic nuclei was observed (Figure 2A,B). Loss of synapses was also investigated as sign of neuronal cell damage. At 10 DIV, primary retinal cell cultures develop abundant synapses, as shown by immunolabelling of synaptophysin, an intrinsic component of synaptic vesicle membranes. The treatment with glutamate induced a decrease in synaptophysin-positive puncta, suggestive of synapse loss. In the presence of citicoline, synaptophysin immunolabeling was similar to control cultures (Figure 2C). Citicoline has been already described to protect against glutamate excitotoxicity in cerebellar granule cells [9] and motor neurons [10]. *In vivo*, citicoline antagonized kainic acid-induced neuronal cell death in the retina, when injected intravitreously [13]. Considering that excitotoxicity plays a crucial role in the pathophysiology of glaucoma [14], our results suggest that citicoline ability to counteract glutamate toxic effect may contribute to slow down the disease progression.

### 2.3. Citocoline Protects against HG-Induced Neurotoxicity

A neuroprotective effect was also detected against HG-induced neurotoxic damage. Primary retinal cell cultures, exposed to daily changes of HG for 96 h, showed a moderate increase in apoptosis, which was reversed in the presence of 100 μM citicoline (Figure 3A,B). Moreover, after the treatment with HG for 96 h, we observed a neurotoxic effect in terms of decrease in synaptophysin expression. Both WB and morphometric analyses showed a reduction in synaptophysin levels. Again in the presence of citicoline, synaptophysin levels were similar to control cultures (Figure 3C–E). Treatment with iso-osmolar mannitol did not induce increase in apoptosis (Figure 3B,F) or decrease in synaptophysin immunolabeling (Figure 3F).

It is now widely recognized that in the early phases of diabetic retinopathy (DR), neurodegenerative changes occur before the alterations of the vascular system, which predominate in the pathologic findings of advanced disease [15–17]. Pursuing neuroprotection is therefore a new therapeutic strategy in the treatment of DR [18,19]. Our results show that, following exposition to HG cell culture medium, citicoline reduced apoptosis, as already described in retinal tissue cultures [8], and reversed synapse loss. Interestingly, downregulation of presynaptic proteins has been described as an early sign of neurotoxicity in DR [20]. The neuroprotection against the effects of HG may again arise from anti-excitotoxic ability of citicoline, since elevated glucose may alter glutamate neurotransmission and calcium homeostasis in primary retinal cultures [21] and dysregulated glutamate metabolism may contribute to neuronal cell damage exposed to the diabetic milieu [22]. Alternatively, it may arise from citicoline antioxidant activity [23], since HG treatment induces an oxidative stress, as shown by increased reactive oxygen species generation [24].

## 3. Experimental Section

### 3.1. Retinal Cultures

All animal studies have been approved by the appropriate ethics committee and have therefore been performed in accordance with the ethical standards laid down in the 1964 Declaration of Helsinki and its later amendments. Primary retinal cultures were obtained from Wistar rat embryos at gestational day 18, as already described [25,26]. After dissociation in trypsin, retinal cells were seeded onto poly-l-lysine-coated cell culture plates or glass coverslips and grown in MEM/FCS (Life Technologies, Monza, Italy), giving rise to a mixed glial-neuronal cell population.

### 3.2. Cell Culture Treatments

To induce excitotoxic apoptotic cell damage, at 8 DIV, cell cultures were treated with 100 μM glutamate for 25 min. Medium was then replaced with fresh MEM/FCS and the cells were fixed after 24 h. In order to recreate the diabetic conditions, primary retinal cultures were treated with HG (30 mM) at 6 DIV for 96 h. Half of the cell culture medium was changed with fresh HG medium every 24 h, in order to keep glucose concentration high. Control cultures were maintained in MEM/FCS with normal concentration of glucose (NG, 5.5 mM). Citicoline (Kyowa Hakko Bio Co., Ltd., Tokyo, Japan) was dissolved in MEM to obtain a 100 mM stock solution. To evaluate its effect on cell survival and differentiation, primary retinal cultures were treated with 10, 100 and 1000 μM citicoline for 96 h. To assess neuroprotective activity, 100 μM citicoline was delivered to cell cultures 24 h before glutamate treatment and 30 min before HG treatment and every 24 h with fresh HG medium. After treatments, retinal cultures were fixed for 25 min in 4% paraformaldehyde in PBS, 0.12 M in sucrose. Controls included retinal cultures exposed to NG or treated with iso-osmolar mannitol (5.5 mM glucose + 24.5 mM mannitol).

### 3.3. Apoptosis Detection

For apoptosis detection, the Terminal transferase-mediated dUTP-biotin Nick End-Labeling (TUNEL) assay was conducted, using the DeadEnd kit (Promega, Madison, WI, USA). Nuclei were stained with Hoechst 33258. Cells undergoing apoptotic cell death were imaged using an Eclipse 80i Nikon Fluorescence Microscope equipped with a VideoConfocal (ViCo) system (Nikon Instruments, Amsterdam, The Netherlands). Apoptosis was expressed as percentage of TUNEL-positive nuclei cells over total cells (at least 500 cells for each coverslip).

### 3.4. Immunocytochemistry

For immunocytochemical characterization, retinal cell cultures were immunolabeled overnight at 4 °C with monoclonal anti-synaptophysin purchased from BD Transduction Laboratories (Franklin Lakes, NJ, USA), monoclonal anti-CRALBP from Santa Cruz Biotechnology (Dallas, TX, USA), rabbit polyclonal anti-GABA from Sigma (St. Louis, MO, USA), monoclonal anti-rhodopsin from Millipore (Billerica, MA, USA). Primary antibodies were revealed with secondary antibodies coupled to Alexa Fluor^®^ 488 or Alexa Fluor^®^ 546 (Molecular Probes, Eugene, OR, USA) (45 min, 37 °C). Nuclei were occasionally stained with Hoechst 33258. Immunostained retinal cultures were observed at the Eclipse 80i Nikon Fluorescence Microscope equipped with a ViCo system (Nikon, Tokyo, Japan).

### 3.5. Morphometric Analysis

Morphometric analysis was conducted as already described [27]. Briefly, in synaptophysin-immunostained primary retinal cultures, images were captured at Nikon Fluorescence Microscope, equipped with the ViCo system and synaptophysin-positive areas were measured inside cell clusters with the ImageJ 1.47v image processing software. At least 8 fields were randomly chosen from two separate coverslips of the same culture. Measures were averaged, to produce a single mean value for each condition in 4 different cultures. Percentages of control samples were arbitrarily set at 100.

### 3.6. Electrophoresis and Western Blot Analysis

After removal of cell culture medium from each well, the cells were washed with ice-cold PBS, lysed in RIPA buffer (25 mM Tris-HCl pH 7.4, 150 mM NaCl, 1% Triton X-100, 0.1% SDS, 1% sodium-deoxycholate, 1 mM sodium orthovanadate, 5 mM sodium fluoride, 1 mM PMSF, Protease Inhibitor Cocktail, from Roche Molecular Biochemicals, Mannheim, Germany) and dislodged using a sterile cell scraper. Homogenates were placed on ice for 30 min and centrifuged at 16,000× *g* for 15 min at 4 °C. The protein concentration of each extract sample was determined using the Micro BCA Protein Assay Kit (Pierce Biochemicals, Rockford, IL, USA). Proteins were separated on 15% SDS-polyacrylamide gel electrophoresis (SDS-PAGE) and transferred to nitrocellulose membranes at 35 V overnight [28]. The membranes were incubated for 1h at room temperature or overnight at 4 °C with the following primary antibodies: polyclonal anti-cleaved caspase 3 (Cell Signaling Technology, Danvers, MA, USA), monoclonal anti-β-actin (Calbiochem Oncogene Research Products, Cambridge, MA, USA). The immunoreactive bands were detected by chemiluminescence, coupled to peroxidase activity (ECL kit, Thermo Scientific, Rockford, IL, USA) and imaged with a Bio-Rad ChemiDoc XRS system (Bio-Rad Laboratories Inc., Hercules, CA, USA).

### 3.7. Statistical Analysis

Data were expressed as mean ± SEM of at least four independent experiments. Data were analyzed by non-parametric Wilcoxon matched pairs or Mann-Whitney *U* tests, since the sample size did not allow assuming a normal distribution. All data were considered significantly different at *p <* 0.05.

## 4. Conclusions

Several studies have been focused on citicoline neuroprotection and various mechanisms have been proposed, including prevention of fatty acid release, promotion of phospholipid synthesis in neuronal membranes, increase in glutathione, enhanced translocation of EAAT2 glutamate transporter in membrane lipid rafts, increase in levels of neuroprotectant sirtuin1, and improvement of glutamate uptake [29–34].

Such an ample and diverse range of mechanisms suggests that citicoline neuroprotection is still far from being elucidated. The most diffuse hypothesis to explain the neuroprotective ability of citicoline assumes that the molecules, following injection or ingestion, are metabolized to cytidine and choline, which may be used by neuronal cells to resynthesize CDP-choline in the plasma membrane. Recently, an alternative hypothesis has been proposed, which presumes that the intact citicoline is the active agent, while choline and cytidine are less active metabolites [35]. This explanation would help clarify why citicoline is efficient in *in vitro* systems, where the drug is not metabolized; in addition, it would also explain why liposomal preparations of citicoline, where the drug is delivered as unhydrolized, intact molecule, are more active than the intraperitoneally or intravenously administered drug, which more easily undergo biotransformation.

*In vitro* experimental models, under conditions relevant to retinal neurodegeneration, may represent a useful system to investigate citicoline neuroprotection. Interestingly, some of the mechanisms that have been described, such as increased translocation of EAAT2 glutamate transporter and improved glutamate uptake, possibly implicate a role of glial cells in the neuroprotection. Our cell culture model, composed of a mixed glial/neuronal cell population, can allow evaluation of the effects of citicoline on different cell types and establish their role in neuroprotection.

## References

[1] Diederich K., Frauenknecht K., Minnerup J., Schneider B.K., Schmidt A., Altach E., Eggert V., Sommer C.J., Schäbitz W.R. (2012). Citicoline enhances neuroregenerative processes after experimental stroke in rats. Stroke.

[2] Putignano S., Gareri P., Castagna A., Cerqua G., Cervera P., Cotroneo A.M., Fiorillo F., Grella R., Lacava R., Maddonni A. (2012). Retrospective and observational study to assess the efficacy of citicoline in elderly patients suffering from stupor related to complex geriatric syndrome. Clin. Interv. Aging.

[3] Cotroneo A.M., Castagna A., Putignano S., Lacava R., Fantò F., Monteleone F., Rocca F., Malara A., Gareri P. (2013). Effectiveness and safety of citicoline in mild vascular cognitive impairment: the IDEALE study. Clin. Interv. Aging.

[4] Parisi V., Coppola G., Centofanti M., Oddone F., Angrisani A.M., Ziccardi L., Ricci B., Quaranta L., Manni G. (2008). Evidence of the neuroprotective role of citicoline in glaucoma patients. Prog. Brain Res.

[5] Parisi V., Manni G., Colacino G., Bucci M.G. (1999). Cytidine-5′-diphosphocholine (citicoline) improves retinal and cortical responses in patients with glaucoma. Ophthalmology.

[6] Rejdak R., Toczolowski J., Kurkowski J., Kaminski M.L., Rejdak K., Stelmasiak Z., Grieb P. (2003). Oral citicoline treatment improves visual pathway function in glaucoma. Med. Sci. Monit.

[7] Ottobelli L., Manni G.L., Centofanti M., Iester M., Allevena F., Rossetti L. (2013). Citicoline oral solution in glaucoma: Is there a role in slowing disease progression?. Ophthalmologica.

[8] Oshitari T., Yoshida-Hata N., Yamamoto S. (2010). Effect of neurotrophic factors on neuronal apoptosis and neurite regeneration in cultured rat retinas exposed to high glucose. Brain Res.

[9] Mir C., Clotet J., Aledo R., Durany N., Argemi J., Lozano R., Cervos-Navarro J., Casals N. (2003). CDP-choline prevents glutamate-mediated cell death in cerebellar granule neurons. J. Mol. Neurosci.

[10] Matyja E., Taraszewska A., Nagańska E., Grieb P., Rafałowska J. (2008). CDP-cholineprotects motor neurons against apoptotic changes in a model of chronic glutamate excitotoxicity *in vitro*. Folia Neuropathol.

[11] Choi D.W. (1988). Glutamate neurotoxicity and diseases of the nervous system. Neuron.

[12] Almasieh M., Wilson A.M., Morquette B., Cueva Vargas J.L., di Polo A. (2012). The molecular basis of retinal ganglion cell death in glaucoma. Prog. Retin. Eye Res.

[13] Han Y.S., Chung I.Y., Park J.M., Yu J.M. (2005). Neuroprotective effect of citicoline on retinal cell damage induced by kainic acid in rats. Korean J. Ophthalmol.

[14] Baltmr A., Duggan J. (2010). N*iz* ari, S., Salt, T.E., Cordeiro, M.F. Neuroprotection in glaucoma—Is there a future role?. Exp. Eye Res.

[15] Antonetti D.A., Barber A.J., Bronson S.K., Freeman W.M., Gardner T.W., Jefferson L.S., Kester M., Kimball S.R., Krady J.K., LaNoue K.F. (2006). Diabetic retinopathy: Seeing beyond glucose-induced microvascular disease. Diabetes.

[16] Lieth E., Gardner T.W., Barber A.J., Antonetti D.A., Penn State Retina Research Group (2000). Retinal neurodegeneration: Early pathology in diabetes. Clin. Exp. Ophthalmol.

[17] Parisi V., Uccioli L. (2001). Visual electrophysiological responses in persons with type 1 diabetes. Diabetes Metab. Res. Rev.

[18] Hernández C., Simó R. (2012). Neuroprotection in diabetic retinopathy. Curr. Diabetes Rep.

[19] Ola M.S., Nawaz M.I., Khan H.A., Alhomida A.S. (2013). Neurodegeneration and neuroprotection in diabetic retinopathy. Int. J. Mol. Sci.

[20] VanGuilder H.D., Brucklacher R.M., Patel K., Ellis R.W., Freeman W.M., Barber A.J. (2008). Diabetes downregulates presynaptic proteins and reduces basal synapsin I phosphorylation in rat retina. Eur. J. Neurosci.

[21] Santiago A.R., Rosa S.C., Santos P.F., Cristóvão A.J., Barber A.J., Ambrósio A.F. (2006). Elevated glucose changes the expression of ionotropic glutamate receptor subunits and impairs calcium homeostasis in retinal neural cells. Investig. Ophthalmol. Vis. Sci.

[22] Stem M.S., Gardner T.W. (2013). Neurodegeneration in the pathogenesis of diabeticretinopathy: Molecular mechanisms and therapeutic implications. Curr. Med. Chem.

[23] Adibhatla R.M., Hatcher J.F., Dempsey R.J. (2002). Citicoline: Neuroprotective mechanisms in cerebral ischemia. J. Neurochem.

[24] Xie B., Jiao Q., Cheng Y., Zhong Y., Shen X. (2012). Effect of pigment epithelium-derived factor on glutamate uptake in retinal Muller cells under high-glucose conditions. Investig. Ophthalmol. Vis. Sci.

[25] Malchiodi-Albedi F., Feher J., Caiazza S., Formisano G., Perilli R., Falchi M., Petrucci T.C., Scorcia G., Tombran-Tink J. (1998). PEDF (pigment epithelium-derived factor) promotes increase and maturation of pigment granules in pigment epithelial cells in neonatal albino rat retinal cultures. Int. J. Dev. Neurosci.

[26] Matteucci A., Cammarota R., Paradisi S., Varano M., Balduzzi M., Leo L., Bellenchi G.C., de Nuccio C., Carnovale-Scalzo G., Scorcia G. (2011). Curcumin protects against NMDA-induced toxicity: A possible role for NR2A subunit. Investig. Ophthalmol. Vis. Sci.

[27] Malchiodi-Albedi F., Paradisi S., di Nottia M., Simone D., Travaglione S., Falzano L., Guidotti M., Frank C., Cutarelli A., Fabbri A. (2012). CNF1 improves astrocytic ability to support neuronal growth and differentiation *in vitro*. PLoS One.

[28] Gaddini L., Villa M., Matteucci A., Mallozzi C., Petrucci T.C., di Stasi A.M., Leo L., Malchiodi-Albedi F., Pricci F. (2009). Early effects of high glucose in retinal tissue cultures Renin-Angiotensin system-dependent and -independent signaling. Neurobiol. Dis.

[29] Hurtado O., Hernandez-Jimenez M., Zarruk J.G., Cuartero M.I., Ballesteros I., Camarero G., Moraga A., Pradillo J.M., Moro M.A., Lizasoain I. (2013). Citicoline (CDP-choline) increases Sirtuin1 expression concomitant to neuroprotection in experimental stroke. J. Neurochem.

[30] Hurtado O., Lizasoain I., Moro M.A. (2011). Neuroprotection and recovery: Recent data at the bench on citicoline. Stroke.

[31] Hurtado O., Pradillo J.M., Fernandez-Lopez D., Morales J.R., Sobrino T., Castillo J., Alborch E., Moro M.A., Lizasoain I. (2008). Delayed post-ischemic administration of CDP-choline increases EAAT2 association to lipid rafts and affords neuroprotection in experimental stroke. Neurobiol. Dis.

[32] Kitada M., Kume S., Kanasaki K., Takeda-Watanabe A., Koya D. (2013). Sirtuins as possible drug targets in type 2 diabetes. Curr. Drug Targets.

[33] Saver J.L. (2008). Citicoline: Update on a promising and widely available agent for neuroprotection and neurorepair. Rev. Neurol. Dis.

[34] Secades J.J., Lorenzo J.L. (2006). Citicoline: Pharmacological and clinical review, 2006 update. Methods Find Exp. Clin. Pharmacol.

[35] Grieb P. (2014). Neuroprotective properties of citicoline: Facts, doubts and unresolved issues. CNS Drugs.

